# Long-term differentiating primary human airway epithelial cell cultures: how far are we?

**DOI:** 10.1186/s12964-021-00740-z

**Published:** 2021-05-27

**Authors:** Zuzanna Bukowy-Bieryłło

**Affiliations:** Institute of Human Genetics PAS, Strzeszynska 32, 60-479, Poznan, Poland

**Keywords:** Primary airway cell culture, Air–liquid interface culture, Conditional reprogramming, ROCK inhibitor, SMAD inhibitor, TGF-β1 inhibitor

## Abstract

**Background:**

Human airway epithelial (HAE) cellular models are widely used in applicative studies of the airway physiology and disease. In vitro expanded and differentiated primary HAE cells collected from patients seem to be an accurate model of human airway, offering a quicker and cheaper alternative to the induced pluripotent stem cell (iPSCs) models. However, the biggest drawback of primary HAE models is their limited proliferative lifespan in culture. Much work has been devoted to understand the factors, which govern the HAE cell proliferation and differentiation, both in vivo and in vitro. Here, I have summarized recent achievements in primary HAE culture, with the special emphasis on the models of conditionally reprogrammed cells (CRC), which allow longer in vitro proliferation and differentiation of HAE cells. The review compares the CRC HAE technique variants (feeder culture or HAE mono-culture), based on recently published studies exploiting this model. The advantages and limitations of each CRC HAE model variant are summarized, along with the description of other factors affecting the CRC HAE culture success (tissue type, sampling method, sample quality).

**Conclusions:**

CRC HAE cultures are a useful technique in respiratory research, which in many cases exceeds the iPSCs and organoid culture methods. Until the current limitations of the iPSCs and organoid culture methods will be alleviated, the primary CRC HAE cultures might be a useful model in respiratory research.

**Plain English summary:**

Airway epithelium (AE) is a type of tissue, which lines the whole length of human airways, from the nose to the bronchi. Improper functioning of AE causes several human airway disorders, such as asthma, chronic obstructive pulmonary disease (COPD) or cystic fibrosis (CF). Much work has been devoted to finding the best scientific model of human AE, in order to learn about its functioning in health and disease. Among the popular AE models are the primary in vitro cultured AE cells collected from human donors. Unfortunately, such human AE (HAE) cells do not easily divide (expand) in vitro; this poses a large logistic and ethical problem for the researchers. Here, I summarize recent achievements in the methods for in vitro culture of human AE cells, with special emphasis on the conditionally reprogrammed cell (CRC) models, which allow longer and more effective expansion of primary human AE cells in vitro. The review describes how the specific chemicals used in the CRC models work to allow the increased HAE divisions and compares the effects of the different so-far developed variants of the CRC HAE culture. The review also pinpoints the areas which need to be refined, in order to maximize the usefulness of the CRC AE cultures from human donors in research on human airway disorders.

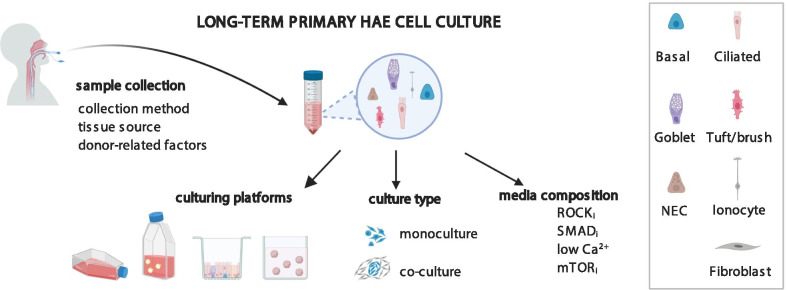

**Video abstract**

**Supplementary Information:**

The online version contains supplementary material available at 10.1186/s12964-021-00740-z.

## Background

Respiratory diseases, both environmentally-induced, such as chronic obstructive pulmonary disease, COPD, or asthma [1], and hereditary, such as cystic fibrosis (CF) or primary ciliary dyskinesia (PCD), affect the major part of present-day societies. Respiratory diseases are one of the most common causes of death globally [2] and pose a large burden to any healthcare system. Accurate diagnosis and efficient therapies of respiratory diseases require in-depth knowledge of their pathophysiology and underlying biology of the airway cell and tissue. Unfortunately, lack of appropriate models of differentiated respiratory epithelium and the insufficient cost-, time- and workload efficiency of the existing models make the basic and applicative studies of pathogenic processes associated with the respiratory diseases, a difficult and cumbersome task.

An effective model of a tissue has to fulfill a range of requirements: it has to be robust and consistent, cost-effective and scalable. Moreover, it has to accurately represent the native tissue, in order to be relevant to the studied disease or condition [3]. Animal models of airway epithelium, including mouse or rat, are a robust source of the well-differentiated respiratory epithelium and thus, are often exploited in studies of respiratory diseases. However, due to a relatively small number of cells acquired from a single animal (e.g. mice or rat), such models are not fully scalable, and using a large number of animals per experiment poses an ethical burden. Moreover, larger animal models such as dogs, pig or cattle, require a considerably more space and have a longer life cycle, making these animal models less cost-effective. However, even the most cost- and time-efficient animal models, such as rodents, do not always fully represent the structure and physiology of the human respiratory epithelium. For example, the composition of airway epithelium in different areas of the respiratory system in mice differs from that in humans [3–5]. Moreover, some animal models might not always fully reflect the human symptoms. For example, in the animal model of PCD caused by lack of DNAH5 protein, mice lacking DNAH5 display hydrocephalus, which is not present in humans with *DNAH5* mutations [6]. The difference between the animal and human models can lead to problems with recapitulating human symptoms in animal disease models, or to false positive results in animal studies, which later lead to therapeutic failures of clinical trials in humans [7]. Thus, animal disease models have to be chosen with care, taking into consideration the similarity in the structure and physiology between the animal and human tissues.

In vitro cultured primary human airway epithelial (HAE) cells seem to be the most adequate model for studies on the functioning of human respiratory epithelium in airway diseases. The successful application of HAE cultures from patients not only limits the use of laboratory animals, in agreement with the animal reduction policies. HAE cultures also advance work on the pathophysiology of the disease, allowing to explain some of the clinical symptoms in the patients, or the disease course [8, 9]. HAE cultures are also essential for the diagnosis of airway syndromes notoriously difficult to diagnose (e.g. PCD) [10]. They are also essential for the development of personalized therapies, such as correction of mutations inducing premature termination codons (e.g. in the cystic fibrosis transmembrane conductance regulator protein, CFTR, in CF) [11, 12]. HAE cultures are also an adequate model to study host-pathogen interactions [13, 14], which has to be remembered in the light of the recent SARS-COV-2 pandemia.

Over the years, many different models of HAE have been developed, ranging from primary airway cell culture, through induced pluripotent stem cells (iPSCs), to immortalized cell lines (see below for details) [7, 9, 13]. However, not all these models fulfill the requirements for a good disease model [3] (see below for details). Here, I summarize recent achievements in the culture of HAE cells, with the special emphasis on the conditionally reprogrammed cell (CRC) models, which allow long-term expansion and differentiation of primary HAE cells, exceeding 4 passages. The review presents available HAE culturing platforms and describes the effects of several compounds, used in CRC HAE methods, on primary HAE cells’ proliferation and differentiation. Other factors affecting the CRC HAE culture success, such as tissue sampling method, tissue origin, presence of the multi-ciliated cells (MCC) in the sample, donor’s lifestyle and used medications are also described. The review also pinpoints the areas that need to be developed further, in order to maximize the usefulness of CRC HAE cultures in airway science.

### *Human respiratory epithelium structure and differentiation *in vivo

**Fig. 1:**
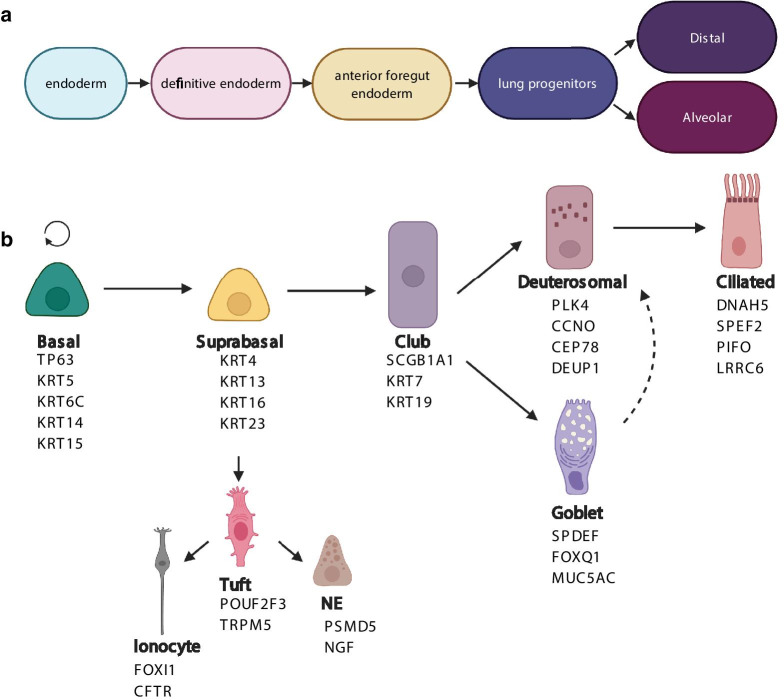
Airway epithelial differentiation pathways. **a** Differentiation of airway epithelial cells during embryonic development. **b** Differentiation of airway epithelial cells after birth (from adult stem cells). Names below the different cell types are the markers of the specific cell types (based on recent human single-cell RNAseq studies [21, 24]). TP63, KRT5, KRT6C, KRT14 and KRT15 are basal cell markers, while supra-basal cells are characterized by expression of KRT4, KRT13, KRT16 and KRT23. Within the mucociliary lineage, club cells are distinguished by expression of Scgb1a1, KRT7 and KRT19, mature goblet cells have expression of SPDEF, FOXQ1 and MUC5AC. The deuterosomal cells express PLK4, CCNO, CEP78 and DEUP1, while mature ciliated cells are distinguished by the expression of DNAH5, SPEF2, PIFO and LRRC6. Suprabasal cells can give rise to tuft cells, which express POUF2F3 and TRPM5. These in turn can differentiate either to ionocytes, expressing FOXI1 or CFTR, or to neuroendocrine cells, expressing PSMD5 or NGF

Conducting airways, which extend from trachea to the proximal end of small bronchioles [15], are lined by pseudostratified respiratory epithelium (PSE). During morphogenesis, respiratory epithelium lining the airways is formed through gradual commitment of the definitive endoderm, to anterior foregut endoderm, lung epithelial progenitors and finally to distal airway or alveolar progenitors [16] (Fig. 1a).

Differentiated PSE is composed mainly of mature secretory (goblet) cells and MCC columnar cells contacting the airway lumen (luminal cells), as well as the basal cells (BCs), which rest on the basement membrane (basal lamina) [17, 18]. Depending on the location in the airways, MCCs, mature mucus secretory cells, and BCs can comprise up to 60%, 10% and 6–30% of PSE cells, respectively [17–19]. Additionally, PSE also contains rare cells, such as tuft/brush cells, neuroendocrine bodies (NEBs) and pulmonary ionocytes [20, 21]. Tuft cells and NEBs primarily serve as chemoreceptors and secrete neuropeptides, which may modulate the tissue microenvironment and innate immunity [20, 22]. On the other hand, recently identified pulmonary ionocytes express high levels of CFTR and thus are probably responsible for the airway surface liquid regulation [23].

BCs, which serve as stem cells of the PSE, constantly replenish the airway epithelium. Upon epithelial airway injury, BCs divide and give rise either to suprabasal cells (a.k.a.luminal progenitors), a common stage in goblet and MCCs differentiation, or directly differentiate into the tuft cells [24]. From tuft cells, ionocytes and NEBs are then formed [24]. Differentiating luminal progenitors give rise to a club cell, another common stage of goblet cells and MCCs differentiation [21]. Club cells may either directly differentiate to goblet cells, or give rise to deuterosomal cells, from which, differentiated MCCs are finally made [25]. Generally, such airway self-renewal is an ongoing, asynchronous process, induced by injury occurring to the different parts of the airway [26].

BCs are abundant in the whole human airways, with lower numbers in smaller airways [17, 21]. BCs are characterized by high expression of TP63, CD44, increased expression of desmosomal proteins (KRT5, -6C, -14 and -15 [27], and low levels of Notch signaling proteins (NOTCH1, NOTCH ICD, DLL-1) [27, 28]. The first BC differentiation stage, suprabasal cell, is characterized by KRT16 and KRT23 expression [21]. Moreover, suprabasal cells may at the same time express KRT13, keratin found typically in the BCs and cells present in the basal layers of the epithelium, together with KRT4, characteristic for more luminal cells [21].

Differentiation of progenitors towards MCCs is associated with the expression of MCIDAS- CCNO-MYB-FOXJ1 genes cascade [21, 29]. Progenitors differentiating towards goblet cells have high expression of SPDEF, FOXQ1 and MUC5AC [21, 26]. Tuft cells, which serve as progenitors of NECs and ionocytes, are characterized by expression of POU2F3 and TRPM5 [22, 24]. After differentiation towards NECs or ionocytes, they acquire expression of PSMD5 and NGF, or FOXI1 and CFTR, respectively [20, 23] (Fig. 1b).

In the absence of BCs, caused by e.g. SO_2_ exposure, upon airway injury, secretory cells are able to co-maintain club cell population, through transdifferentiation to MCCs [4, 21]. Also other cell types of PSE, such as neuroendocrine cells and myoepithelial cells from the submucosal glands, can serve as facultative progenitors in PSE [4]. In contrast, MCCs present in PSE are terminally differentiated and are not able to divide and yield progenitors [21].

### Primary airway epithelial cell culture

First reports on the culture of airway epithelial cells come from the beginning of the 1980’s. However, at that time, the HAE cell propagation and cell differentiation methods were inefficient and technically challenging, thus the use of these methods was limited [30]. Gradually, increased knowledge about the chemical and mechanical factors stimulating HAE cells to propagate or differentiate led to the improvement of the culturing methods, development of dedicated growth media and increased popularity of primary HAE cell cultures [31–34].

In vivo, cellular differentiation during homeostasis is an ongoing, asynchronous process [26]. However, in vitro, the cell expansion and differentiation steps are separated, supported by media containing culture-stage specific supplements (e.g. adequate Ca2 + or retinoic acid, RA, levels) [30, 35]. Also mechanochemical conditions (e.g. contact with atmospheric air, and/or specific stiffness of the substrate, the cells grow in), and the composition of the cellular microenvironment (presence of the extracellular matrix, ECM, molecules), significantly influence the growth and differentiation of airway epithelial cells in cell culture [36].

With time, several culture platforms were developed, such as air–liquid interface culture (ALI) or adherent-suspension culture [31, 37, 38]. Each of these culturing platforms requires stage-specific media and supplements. These can either be prepared in the lab, according to the developed protocols [33, 39], or acquired commercially (BEGM, UltroserG, Epithelix). Currently, culture of primary HAE cells is a frequently used model for research on tissue development, inborn or acquired airway diseases and host–pathogen interactions.

### Advantages and limitations of traditional primary AEC culture

The most important advantage of the primary HAE cell culture from patients, in the context of studying the pathogenesis of the inherited respiratory diseases, is that they provide a source of respiratory cells already containing genetic modifications, without the need to genetically modify the cells in the laboratory. This also encompasses epigenetic modifications—it has been shown that cultured primary airway cells from COPD patients reflect epigenetic DNA changes and replay epithelial reactions to IL-13 [9, 40]. Also in vitro differentiated cells from asthma patients retain characteristics of the distorted airway epithelium in culture, which is important for the studies of environmentally-induced airway disorders [41, 42].

In contrast, iPSCs often lose epigenetic marks [7, 43]. They are also tumorigenic and show genetic instability, which is an important obstacle in the gene therapy and tissue regeneration approaches [42, 44, 45]. Moreover, high costs and long-term derivation of the iPSCs into airway progenitors make the iPSC model less favorable, compared to the primary HAE models.

Primary HAE cultured cells are also a better model than immortalized airway cell lines. Although immortalized airway cell lines have a long proliferative lifespan, are cost-effective and show consistent results (as usually are derived from one donor), the immortalization may reduce the ability of cells to differentiate. In effect, the structure and physiology of airway cell lines do not always reflect the structure or physiology of the native epithelium. The airway cell lines may  lack, for example, mucociliary differentiation, barrier formation or expression of specific receptors required for the virus infection [42, 46]. This makes the airway cell lines less suitable for studies of airway disorders related to cilia (e.g. PCD), mucus (e.g. CF), or viral entry [46]. However, cell lines still can be used for studies requiring the presence of polarized epithelium, including innate immunity, ion physiology or therapeutic approaches [47, 48].

Unfortunately, there are also several disadvantages of the traditional primary HAE culture, which for some time have prevented a broader use of this type of culture in the airway cells research. The main and most significant drawback of primary HAE cells is their limited ability to efficiently proliferate and differentiate in vitro for a larger number of passages. Under normal circumstances, primary HAE cells are able to efficiently divide during only for 3–4 population doublings[49, 50]. After that time, the cells start to divide slower, and gradually lose the ability to form cilia, produce mucus or express tight junction proteins, [50–52]. Obtaining sufficient number of differentiated cells in culture is therefore very difficult, especially in light of the limited availability of tissues/cells (acquired from biopsies or brushings). Moreover, the primary HAE cells may not differentiate very well after cryopreservation, which limits the possibility of later use of stored patients’ samples [53]. In effect, experiments, which require long-term culturing, such as genetic modifications of primary HAE cells, when performed under regular culture conditions, without the addition of factors prolonging the cells’ lifespan, represent a cumbersome and time-consumming task.

Other disadvantages of primary airway epithelial cultures is the frequent lack of consistency or robustness [42]. This might be due to the sampling method, intrinsic differences between the donors (more on this below), but also type of the culture and media used. All these disadvantages contributed to a not very frequent use of the primary HAE cell cultures. This has changed in the recent years, especially after the development of the CRCs cultures.

### Culturing platforms for primary HAE cell culture

**Fig. 2:**
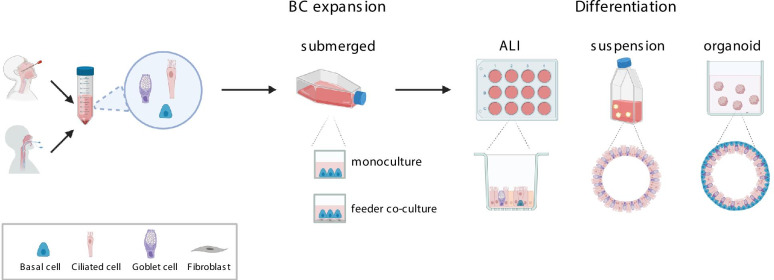
Existing culturing platforms for HAE expansion and differentiation. ALI – air–liquid interface culture; BC- basal cell

Different culture platforms exist to culture and differentiate primary HAE cells. One of the oldest cell culture types is the adherent 2D culture, where the HAE cells are cultured as a single layer, submerged with growth medium. The adherent 2D culture promotes an undifferentiated HAE phenotype, thus it is mostly used for the expansion of HAE BCs (Fig. 2). To improve the adhesion of the HAE cells to the culturing vessels, thin collagen coating or a thicker layer of collagen gel can be used [33, 37].

Several platforms have been developed, which support successful in vitro mucociliary differentiation of HAE cells (Fig. 2). The presently most common differentiation platform is the air–liquid interface (ALI) culture, where dissociated HAE cells are first expanded in submerged culture and then differentiated on a porous membrane insert [33, 54]. After seeding to culture inserts, HAE cells are cultured submerged in the medium, as the medium is added both to the insert inside (apical chamber), and to the well below the insert (basolateral chamber). Upon confluency of the cellular monolayer, differentiation medium is changed to a specific ALI differentiation medium, which is added only basolaterally, and the apical chamber of the insert, together with the cell layer, is exposed to the atmospheric air [33]. During differentiation, the cells in ALI platform develop a typical PSE structure, with non-dividing basal epithelial cells staying close to the insert membrane, and the MCCs and goblet cells oriented towards the air [33] (Fig. 2). The single layered PSE structure allows the ALI cultures to be easily used for the analyses of ion/ drugs transport and measurements of the transepithelial resistance (TEER). However, the differentiation in ALI is sensitive to culture conditions, changes in air humidity, and the differentiation process depends much on the amount and quality of the HAE cells seeded [32]. Differentiated HAE cells can also be obtained in 3D culture, when groups of the expanded HAE BCs are cultured in suspension culture or embedded in ECM matrix [34, 38, 55, 56]. The suspension differentiation of HAE cells has been the basis of the adherent-suspension culturing method, developed by Jorissen [34]. In the adherent-suspension method, cells are first cultured adhered to a collagen gel [34]. Upon confluence, collagen gel layer is digested, and the sheet of confluent cells is transferred to rotating suspension flasks for differentiation. During suspension culture, within 15 days, cell sheets close into spheres (spheroids), which contain goblet and MCCs on the spheroid outer surface (Fig. 2). Differentiation of primary HAE cells in the 3D suspension culture proceeds faster than in ALI [34, 54, 57], however, the adherent-suspension culture method has a limited scalability, as the primary HAE cells grown on collagen gel layer cannot be dissociated to be further expanded  in another vessel.

Moreover, the differentiation of the spheroids in the suspension culture is more asynchronous and depends on the size of the spheroid in question [34]. Some studies have also shown, that differentiated spheroids might not contain BCs, which makes the spheroids grown in suspension not able to self-renew [58].

An alternative method of culturing and differentiating HAE cells in 3D is the recently developed organoid culture [55]. Dissociated primary HAE cells are embedded in a 3D ECM matrix in the presence of media containing tissue self-renewal promoting compounds known from the stem cell research (noggin, inhibitors of the Bone Morphogenetic Protein, BMPi or ROCK proteins, ROCKi, etc.). Upon embedding in the ECM matrix, primary HAE cells start to divide and self-organize to form aggregates, which gradually differentiate into sphere-like organoid structures. In the organoid structures, goblet and MCC cells are facing towards the organoid lumen and the basal stem cells face the outer surface of the organoid (Fig. 2). Due to the presence of basal epithelial stem cells, the organoids are able to self-renew and the organoid cultures can be efficiently proliferated through organoid disruption for a long-time (> 1 year) [59, 60]. However, the 3D structure of the organoids make this culturing platform not suitable for some forms of analyses, e.g. ion function measurements by TEER. Moreover, the size of the organoids is difficult to control, which makes it less suitable for high-throughput analyses and not as reproducible as the ALI culture [3, 44].

## CRC models of HAE

The recent development of CRC technique, which allows prolonging the limited lifespan of the cultured cells, revolutionized the primary HAE cell culture. The CRC approach exploits the use of inhibitors of the Rho-associated coiled-coil–containing protein kinases 1 and 2 (ROCKi) [28, 61]. Rho kinases are small GTPases, which play role in cell–cell adhesion, cell migration, differentiation, apoptosis and proliferation [61].

One of the known ROCKi, Y-27632, has been shown to increase survival of embryonic stem cells (ESCs) in vitro [62]. ROCKi Y-27632, added to the difficult-to-proliferate keratinocytes, traditionally cultured in the presence of lethally irradiated mouse fibroblast feeder 3T3-J2 cells, rapidly changed keratinocyte  morphology and induced robust proliferation, providing a nearly infinite source of undifferentiated keratinocytes [63]. Once the ROCKi and the feeder cells were removed, keratinocytes could later be efficiently differentiated, hence the term ‘conditionally reprogrammed cells” (CRCs) was coined. Further experiments have shown, that this mechanism is effective also in other types of non-keratinocyte epithelia, including HAE cells [28, 61, 64] (Table 1).

**Table 1:** Recent studies exploiting CRC HAE co-culture method with feeder cells

CRC HAE culture with feeder cells							
Publication	Cell type, sample type, age group	Feeder cells? /Cell stasis factor	Expansion	Differentiation	Culture length:	Major findings:	Limitations
Suprynowicz [28]	HTE epithelium	Yes, IR	Submerged culture, KSFM or F-medium, 10uM Y27632; Vessels: no coating	ALI culture, Until confluence- CELLnTEC CnT57 medium + 10 μM Y-27632 Later: CELLnTEC CnT-02–3D differentiation medium, 14 days	Passage number N/A, forms cilia in ALI	1. Stable karyotype, non-tumorigenic 2. Rapid and reversible induction of cell divisions 3. Maintenance of lineage characteristics	Unknown maximal number of passages for HAE culture
Butler [66]	HBE epithelium (biopsies)	Yes, IR or mitomycin C	Submerged culture: DMEM- F12 (3:1 ratio), 5% FBS, 5 uM Y-27632, hydrocortisone (25 ng/ml), epidermal growth factor (0.125 ng/ml) insulin (5 mg/ml), 0.1 nM cholera toxin, amphotericin B (250 mg/ml) and gentamicin (10 mg/ml), pen-strep (1x) Vessels: no coating	Differentiation: suspension culture (tracheospheres) or ALI	Expansion for > 3 months (split ratio 1:5), cells expanded for > 6 weeks can differentiate (tracheospheres, ALI)	1. CRC cells are non-tumorigenic and react to contact inhibition 2. Cell yield sufficient for tissue engineering/ regeneration (> 1 × 10^7^ cells obtained in < 4 weeks)	n/a
Reynolds [27]	HNE brushings of healthy adult donors	Yes, IR	Submerged culture: DMEM:F12 (3:1) + 7,3% FBS, hydrocortisone/EGF mix, Cholera toxin 8,6 ng/ul, Adenine 24 ng/ul, Insulin 10ug/ml Vessel coating: rat tail collagen type I (30 µg/ml) Conditions compared with regular BEGM culture	ALI culture, organotypic raft cultures or 3D tracheospheres in Matrigel	CRC expanded HNE cells could be differentiated at P4, in contrast to BEGM-expanded HNE cells.	1. Increased clone formation 2. Increased cell yield compared to BEGM (~ 400 fold) 3. Mechanism of Y-27632 action: (a) promotion of the basal cell phenotype; (b) stimulation of basal cell–specific cell–cell and cell–ECM interactions; and (c) suppression of differentiation to an airway luminal cell phenotype	1) For some donors—almost no MCC visible (MCC number: from < 10% to ~ 40%); 2) Limitated proliferative lifespan of HNEs expanded in CRC or BEGM, visible already after 2 passages (less pronounced in CRCs)
Wolf [65]	HNE and HBE cells of healthy donors at different ages (from newborns to adults)	conditioned medium from IR fibroblasts	Submerged culture: BEGM medium conditioned by 72 h incubation with NIH-3T3 fibroblasts irradiated with 30 Gy and frozen in aliquots. Conditioned BEGM mixed with fresh BEGM (3:1 v/v); supplemented with 10 µM Y-27632 Vessels coating: human placental collagen type IV	ALI culture; Conditioned BEGM medium + Y-27632 to the basolateral compartment only	~ 13–14 PDs within 30 days (split ratio 1:4)	1. Increased proliferation and extended proliferative lifespan 2. Maintenance of lineage characteristics after multiple passages, 3. Physiological airway responses to dsRNA (induction of antiviral genes, IFN λ1 production, inflammatory and remodeling responses)	1) Weak differentiation at ALI for some donors; 2) Not sure whether the anti-viral responses observed in CRCs are identical to the in vivo HAE responses (CRCs induction changes expression of some inflammatory genes)
Brewington [57]	HNE samples from healthy or CF patients (curettage, from newborns to adults)	YES, IR (commercially available, frozen)	Submerged culture: DMEM/F12 + 10% FBS, cholera toxin (0,01 mg), EGF (0,04ug), HC (0,4ug/500 ml), Adenine (24 mg), Y-27632 (3,2 mg) + antibiotics Vessel coating: VitroCOl	Suspension culture (spheroids): DMEM/F12 + Ultroser G, 20 ml Fetal Clone II, Bovine Brain Extract, Transferrin, epinephrine, ethanolamine, insulin, HC, RA, triiodothyronine, phosphorylethanolamine	spheroids differentiate within 10 days	1. Faster differentiation of spheroids than ALI cultures 2. Spheroid swelling is a quick and robust assay for CFTR activity	1) Short availability of spheroids for analysis 2) No information on the differentiation capacity beyond passage 2 3) Preclinical utility not fully clear (drug testing in CF patients?)
Peters- Hall [69]	bronchial biopsy	YES, IR	Submerged culture: P0-P3: BEGM, 0.5 ng/ml EGF, 2% O_2_ P3 -5: (CRC culture); BEGM, 5% FBS, 10uM Y-27632, no coating, 2% O_2_ Vessel coating: P0-P3: Porcine gelatin, P3-P5: no coating	ALI culture, P5 + (Transwells, expansion): BEGM + 10uM Y-27632, no coating, 21% O_2_ P5 + (ALI): BEGM, 0.5 ng/ml EGF, 0.11 mM CaCl2, 0.1 mg/ml BPE, 21% O_2_	up to 47 PDs (passage 15)	1. Increased ciliation of MOD CRCs at P5 and P10 (~ 33–38%) 2. CFTR activity probably retained at later passages 3. Increased HBECs lifespan allowed CRISPR-Cas9 gene edition 4. Mod CRC conditions reduce cellular stress in HBECs from non-CF donors and prevent premature cellular senescence	Requires various media, complicated procedure

The increased proliferation of HAE cells in the CRC culture massively affected the culture yield [27, 28, 65], with ~ 20-fold increase in cell numbers within 96 h [28]. The high replicative potential of CRC HAE culture, starting from a nasal brushing, could yield in less than 4 weeks cell numbers sufficient to cover a human trachea regeneration scaffold (> 1 × 10^7^ cells) [66]. The increased proliferation did not cause any negative effects on the cultured CRCs—the CRCs maintained a stable karyotype, the contact inhibition ability and were non-tumorigenic [28, 66]. Importantly, the CRC conditions also promoted increased transduction and electroporation [61, 67], without negative effects on HAE cell proliferation or differentiation [61, 68]. The increased proliferation of HAE cells in the CRC culture supported single cell cloning and allowed antibiotic selection of the transduced cell population [28]. This opened the possibility of efficient genetic modifications of primary HAE cells using reporter plasmids [61] or Cas9 nucleases [67, 69]. ROCKi also allowed efficient reinitiation of the HAE cultures after cryopreservation [61]. Generally, addition of ROCKi and feeder cells made primary HAE cultures a more complete and efficient tissue model for airway studies.

However, the CRC HAE conditions with the use of feeder cells (CRC HAE co-culture) were not fully universal and applicable to all types of experiments. For example, it has been shown, that the CFTR function can decline with subsequent passages of the CRC HAE co-culture [70]. To remove that limitation, CRC HAE co-culture at reduced oxygen concentration (2% O_2_-7% CO_2_-91% N_2_ mixture) has been explored [69]. The reduced oxygen conditions not only allowed to obtain differentiated HAE cells with functional CFTR reactions even at P10, but also limited cellular stress, extending the proliferative lifespan of primary HAE cells above 100 PDs, compared to the regular CRC HAE co-culture conditions [69]. In addition, upon differentiation in ALI, HAE cells expanded in low-oxygen CRC co-culture generated PSE containing more MCC cells, compared to regular BEGM and standard CRC HAE co-culture conditions [69] (Table 1).

**Fig. 3:**
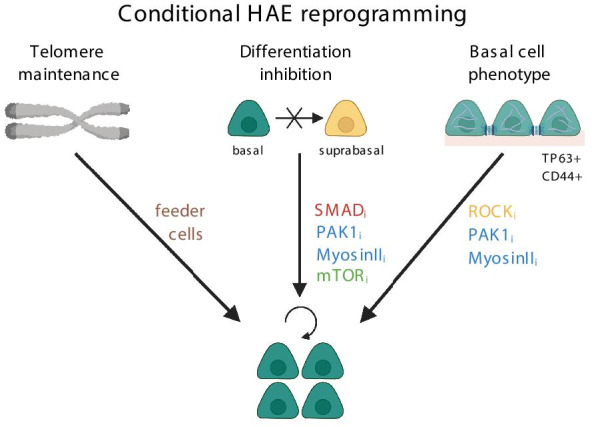
Mechanisms of conditional HAE reprogramming allowing proliferation of basal cells. ROCKi – ROCK inhibitor, PAK1i – PAK1 inhibitor, Myosin IIi- inhibitor of Myosin II, SMADi – inhibitor of SMAD, mTORi – inhibitor of mTOR pathway

Addition of ROCKi to the primary HAE cells quickly induced the adult stem cell phenotype (Fig. 3), associated with increased expression of stem cell markers p63 and CD44, upregulated desmosomal adhesion proteins typical for BCs (upregulated: Krt10, desmoplakin, claudin 10, cadherin 3). ROCKi also inhibited the transition to the luminal airway progenitor phenotype, by repressing signaling pathways (Notch 1 signaling, Notch 1 receptor, Notch ICD, DLL-1), cell–cell interaction molecules (claudin10,11 and occludin) and protease genes (MMP 2,9,14 and 28) typical for the luminal progenitor cells [27, 28]. This led to undifferentiated HAE cell state and longer BCs proliferation [27, 28] (Fig. 3).

The high replicative potential of HAE cells obtained using CRC co-culture method was sufficient to generate HAE cell numbers useful in tissue regeneration [66]; however, the CRC co-culture conditions contained animal origin components, which potentially put a risk to the patient during the transplantation procedures. The increased knowledge of factors influencing airway epithelial cell differentiation allowed modification of the CRC HAE culture method, to avoid  the use of 3T3 murine feeder cells (CRC HAE monoculture method) (Table 2).

**Table 2:** Recent studies exploiting feeder-free methods of CRC HAE culture

Feeder-free CRC HAE culture								
	Publication:	Cell type, source, age	Feeder cells?	Expansion:	Differentiation:	Culture length:	Major findings:	Limitations
ONLY ROCKi	Horani [61]	HTE and proximal HBE cells from surgical specimens, expanded and cryopreserved at P1	NO	Submerged culture: DMEM/F-12 with 15 mM HEPES, 4 mM L-Glutamine, 3.6 mM NaHCO_3_, 100 U/mL penicillin, and 100 mg/mL streptomycin with supplements (10 mg/mL insulin, 5 mg/mL transferrin, 0.1 mg/mL cholera toxin, 25 ng/mL EGF, 30 mg/mL BPE, 5% FBS (v/v)) Vessel coating: rat tail collagen type I (50 mg/ml)	In ALI, mTEC/Basic Medium with 2% NuSerum and retinoic acid [54]	Highest proliferation at 5 µM Y27632. ~ 25% cilia in hTEC	1. Y27632 increased cell proliferation, efficiency of lentiviral transduction (up to 80%), and facilitated antibiotic selection of transduced cells	Decreased number of goblet cells during ALI (0–19 Muc5AC + cells/ 100-power visual field)
	Jonsdottir [68]	Fresh HBE cells (bronchoscopy or surgical samples), adult patients	NO	Submerged culture: BEGM medium with 10uM Y-27632 Vessel coating: Collagen Type IV	ALI, LHC:DMEM medium + supplements [33] Vessel coating: Collagen Type IV	Cells used until P4	1. Lentiviral suspension transduction method established (15 -70% efficiency, depending on the virus titer) 2. Addition of ROCKi slightly decreased this efficiency (5%–10% lower), but allowed selection of cells after transduction	Not all constructs were equally effective—careful evaluation and testing of the viral constructs is required
OTHER SUPPLEMENTS/ INHIBITORS	Mou et al. [71]	fresh human trachea, bronchi, BAL, or induced sputum	NO	Submerged culture: Different media (with/without 5–10 mM ROCKi, 0.5–1 mM A-83–01, 0.5–1 mM DMH-1, and 1 mM CHIR99021, separately or in various combinations). Media tested: SAGM (Lonza), HTEC (You and Brody, 2013), BEGM (Fulcher et al. 2005), LHC (Life Technologies), LHC-9 (Invitrogen), and AECBM (from ATCC and PromoCell) Vessel coating: laminin-enriched 804G-conditioned medium	ALI: Pneumacult ALI medium	Cell divisions until P25-P30 (PD > 40) Goblet cell differentiation until P25, but efficient ciliation until P10 Physiological CFTR reactions—until P8 TEER – stable	1. Expansion possible up to 25 passages (PD 40) 2. Expansion is efficient (from 1–20 *10^3^ cells to ~ 1 × 10 ^15^ cells at P10—in 50 days) 3) BAL or induced sputum samples (< 2000 cells) expanded to 10^9^ or 10^10^ within 1 month	Resemblance to native epithelium decreases with time (CFTR reactions only until P8, mucociliary differentiation until P10) 3) Culture from induced sputum samples less effective than from BAL
OTHER SUPPLEMENTS/ INHIBITORS	Zhang et al. [72]	HBE cells	NO	Submerged culture: KSFM with 1 µM A83-01, 5 µM Y-27632, (KSFM + A + Y) Vessel coating: collagen I	ALI, Filter seeding in EpiX + 1.5 mM CaCl2. After confluence, basolaterally Pneumacult-ALI medium	EpiX medium supports efficient HBECs expansion and differentiation until 45–60 PDs (P12–P16)	1. Increased cell yield (> 50 mln cells from CF patients in 3–4 weeks; > 1 * 10^6^-fold increase) 2. Retained genome integrity, no tumorigenicity 3. Cells have low stress levels 4. Retained CFTR response > 30 PDs (reduced, but exists); 5. Single cell cloning possible	Large cells accumulated slowly over passages and eventually the majority of the population appeared senescent or differentiated
	Lu et al. [74]	tracheal neonatal aspirates (< 100 epithelial cells/ sample)	NO	Submerged culture: SAEG with A-8301 (1 μM) and Y27632 (5 μM), rapamycin (5 nM) Vessel coating: N/A	ALI Filter seeding: growth medium, Pneumacult-ALI and DMEM with 4-(2-hydroxyethyl)-1-piperazineethaNesulfonic acid (2:1:1 ratio) 12 h after filter seeding: complete Pneumacult-ALI medium (submerged, ALI from day 1)	Expansion for at least P15 (40–50 PDs) Cells retain BC markers (NGFR, PDPN) Differentiation until P16	1. Efficient generation of cultures from neonatal tracheal aspirates 2. Efficiency of mucociliary differentiation comparable to other methods	Abrupt halt of proliferation at the end of the culture– probably due to telomere erosion
	Koh et al. [67]	HBE cells, from leftover transplant	NO	Submerged culture: BEGM + 10 uM Y-27632 Vessel coating: human placental collagen	Medium: LHC:DMEM mixture with supplements [33]	N/A	Double nucleofection allowed up to 100% targeting without antibiotic selection	

Mou and coworkers have reported that SMAD/TGF-β signaling is active in differentiating HAE cells and absent in basal HAE cells [71]. Addition of two inhibitors of SMAD pathway effectors TGF-β and ALK2 (A-83-01 and DMH-1, respectively), to the growth medium containing ROCKi Y-27632 allowed efficient expansion of primary HAE cells. This was possible through the inhibition of the BC differentiation towards luminal progenitors cells [71] (Fig. 3). The HAE expansion persisted until HAE passage 25–30 (~ 80 PDs), without induction of telomerase (hTERT) expression. Thus, upon telomere erosion, cells abruptly stopped propagation. Despite efficient proliferation, the characteristics of the differentiated airway epithelium were not consistent—MCC cell differentiation declined after P10, but physiological CFTR reactions were retained only until P8 [71] (Table 2).

The influence of SMAD/ROCK inhibitors on airway BCs' proliferation was also confirmed by Zhang and coworkers, who identified SMAD and ROCKi as independent factors in a screening assay of small molecules able to affect  HAE BCs proliferation [72]. Further optimization of the SMAD/ROCKi method by the use of inhibitors of downstream ROCK effectors, PAK1 (p21- associated kinase) or Myosin II, and reduction of the differentiation-inducing Ca^2+^ levels in the culture medium (EpiX medium) increased BCs’ proliferation even more, mainly through the inhibition of the differentiation towards basal luminal progenitors (Fig. 3). The inhibition of the ROCK-SMAD-PAK1/Myosin axis allowed efficient cell proliferation of primary HBECs until 45–60 PDs (12–16 passages) [72]. Generally, this particular CRC method lead to over 1,000,000 fold increase in HAE cell numbers, without the use of the feeder cells. However, cellular senescence was not avoided, as large cells increasingly accumulated from passage to passage [72] (Table 2).

Mammalian target of rapamycin (mTOR) pathway is important for the proliferation of airway BCs, however the activation of mTOR pathway also induces HAE differentiation [73] (Fig. 3). Under repeated or chronic damage, constant mTORC1 activation leads to the reduction in the numbers of the airway BCs and the regenerative capacity of the airway [73]. Use of mTOR inhibitor (mTORi), rapamycin, combined with SMAD and ROCK inhibitors (A-8301 and Y-27632) successfully supported expansion and growth of human neonatal tracheal epithelial cells from aspirates [74], which contained very low initial number of cells (< 100 cells/aspirate). The triple inhibition supported growth of expanded cells for at least 15 passages (~ 45 PDs). During the culture, cells consistently expressed BCs markers and efficiently differentiated even at passage 16 [74]. Rapamycin did not induce or inhibit genes involved in oxidative stress, epithelial—mesenchymal transition, or cellular senescence (Table 2).

### Media and vessel coating for HAE proliferation

Generally, most of the CRC HAE feeder co-cultures use the original F-medium for keratinocytes published by Liu et al., but some studies have also successfully used BEGM medium for that purpose [64, 69]. In some cases, BEGM conditioned by incubation with lethally irradiated feeder cells was added to the HAE cell monoculture [65]. However, the effect of such conditioned medium on cell proliferation was not as effective, as direct co-culture with irradiated feeder cells.

Studies have shown that a range of growth media can be used for the CRC HAE monocultures, (F-medium, BEGM, SAGM, HTEC or LHC-9 media), as long as the media contain the appropriate inhibitors inducing the CRC state (ROCKi, SMADi, mTORi, PAK or myosin II inhibitors) [61, 67, 68, 71, 74]. However, the largest HAE expansion and best long-term effects were observed in the KSFM medium, which contains low levels of calcium, additionally preventing the differentiation of the BCs [72]. Regarding the culture vessels, most of the CRC HAEs studies continue to use culture vessels coated with basement matrix molecules, such as collagen (type I or type IV) [27, 57, 61, 65–68, 72]. Rarely, solutions containing laminin [71, 74], or porcine gelatin [69] were used. For HAE differentiation, most published studies have also used ALI platform, with classic home-made [33, 54], or commercially available HAE differentiation media (BEGM, Pneumacult-ALI, CELLnTEC, Vertex ALI, Epithelix + 1,5 mM Ca^2+^) [57, 65, 69, 71, 72, 74].

### Experimental considerations for the CRC HAE culturing methods

In general, both CRC HAE regimens are rather similar—they robustly enhance HAE proliferation, leading to an increased cellular yield. The expanded HAE cells most often effectively generate differentiated HAE containing MCC and goblet cells and displaying ion function (e.g. CFTR activity). In most of the cases, applied CRC HAE culturing methods do not have a large influence on the differentiation potential of expanded basal cells, and the exact numbers of MCC and goblet cells in the differentiated epithelium depend rather on the donor, the cellular passage and the type of medium used for differentiation (compare Tables 1, 2). In this context, the only exception was the method by Peters-Hall et al., where the airway BCs were expanded at reduced oxygen pressure (2% oxygen) [69]. Differentiation of such BCs in ALI yielded differentiated epithelium with increased amount of ciliated cells compared to the normal oxygen conditions. Low oxygen levels are known to prevent differentiation of MCCs and some studies show that hypoxia may also promote the secretory (goblet) cell phenotype [75]. Increased ciliation after the differentiation of BCs expanded under hypoxic conditions may have been a result of a larger pool of club cells, common stage of goblet cell and MCCs differentiation. Alternatively, an improved ability of the BCs to differentiate into different lineages (increased stemness) is responsible for this effect.

Due to the logistics related to preparation of the growth-inhibited feeder cells (seeding, irradiation/ mitomycin treatment), the workload in the CRC co-culture method is much higher than in the CRC monoculture [76]. Although the amount of work can be reduced by the use of commercially available frozen irradiated feeder cells [27], the work related to the differential cell trypsinization of the feeder and HAE cells still remains. To reduce this workload, some studies have tested the possibility of using growth medium conditioned by the incubation with irradiated feeder cells, but this approach was not as efficient as the direct co-culture with feeder cells [65]. Moreover, another drawback of the CRC co-culture method is the potential contamination of CRC-expanded HAE cells with animal-origin feeder cells, which makes the co-culture CRC HAE method not suitable for human therapy [71].

The CRC HAE monoculture method does not suffer from that limitation and can be used in human therapeutic applications. Compared to the CRC HAE co-culture, the CRC HAE monoculture uses more chemically-defined growth media. Moreover, the expanded HAEs have slightly longer proliferative lifespan (45–60 PDs) and display slower decline in CFTR activity with passages (p8 vs p5 in the CRC HAE co-culture) [50, 71]. However, recent results indicate, that the CRC monoculture-expanded HAE cells might display different physiology, compared to HAE cells expanded using the co-cultured CRC method [77]. This includes reduced beating frequency of airway cilia and the lower ion currents than the ALI-differentiated HAE cells,  expanded using the co-culture CRC method [77]. In that aspect, the monoculture CRC method might not as accurately represent the native epithelium as the co-culture CRC HAE method.

**Fig. 4:**
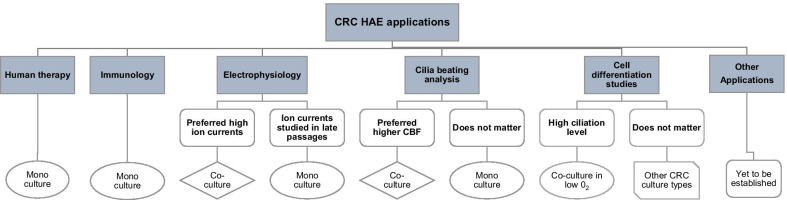
Choice of the CRC HAE culturing method depending on the experimental application. CBF – ciliary beat frequency

Therefore, the choice of the particular CRC HAE method should depend not only on the available equipment and workforce, but also on the particular experimental application of the CRC HAE cultures (Fig. 4). While benefits of the CRC HAE monoculture in immunology and human therapy applications are obvious, the choice of the particular CRC HAE culturing method for studies of ciliary beating or airway ion currents will depend on the preferred experiment time-frame or CBF intensity (Fig. 4). The CRC HAE proliferation method most suitable for other experimental applications is yet to be determined (Fig. 4).

## Other factors affecting primary HAE culture success

### Sampling method

Apart from the media composition, there are other factors, which influence the success of primary airway epithelial culture. As only 10–30% of the collected cell number are the BCs[17, 19], successful culture should contain the highest possible number of airway cells, in the best possible condition. This depends very much on the effectivity and quality of the tissue sample, but also the donor’s infection history, received medications and lifestyle factors.

The number and quality of cells in the epithelial sample are most important for the successful initiation of the airway epithelial cell cultures. The highest initial number of primary HAE cells can be obtained from tissues obtained via resection, transplantation or autopsy. However, these procedures are highly invasive, thus the supply of the tissues can be limited. Alternative collection methods, such as nasal or (nonbronchoscopic) bronchial brushings, are simple, quick, minimally invasive and can be performed even at the young age [49, 50]. Brushings can also yield relatively high initial cell numbers (nasal brushing: up to 1.5 mln cells, bronchial brushings 2.5–3.5 mln cells/ two brush passes [19, 78], leading to a relatively high culture success rate (Table 3) [19, 49, 50].

**Table 3:** CRC HAE culture cell yield and success rate depending on the tissue collected

Sampling method	Age group (condition)	Initial cell number	Subculturing method	Success rate in propagation	References
Induced sputum	Children	< 2000 cells/ portion	CRC HAE mono (Rho/SMAD inhibition)	20% in CF patients	Mou et al. [71]
Tracheal aspirate	Neonatal	< 100 cells/ aspirate	CRC HAE mono (Rho/SMAD/mTOR inhibition)	40% from single sample, 80% if multiple samples/ patient	Lu et al. [74]
BAL	Neonatal	< 2000 cells/ portion	CRC HAE, mono (Rho/SMAD inhibition)	100% (multiple samples/ patient)	Mou et al. [71]
Nasal brushing	N/A (CF or healthy)	0.4 to 1.5 million viable cells	Traditional ALI culture	Culture initiation: CF patients—66%; healthy—85% Differentiation in ALI: 100%	Schogler et al. [78]
Bronchial brushing	N/A	0.045 – 0.2 mln cells/ brush	Indirect CRC HAE co-culture: HAE cells cultured with conditioned fibroblast medium with ROCKi	N/A	Wolf et al. [65]
Bronchial brushing (non-bronchoscopic)	Children (healthy or asthmatic)	~ 2,67 mln cells/ 2 brush passes	CRC HAE co-culture, direct	N/A	Martinovich et al. [50]

*BAL* Bronchioalveolar lavage, *CRC HAE mono* conditionally reprogrammed human airway epithelial monoculture (without feeder cells), *CRC HAE co-culture* conditionally reprogrammed human airway epithelial co-culture (with feeder cells)

In contrast, isolation of HAE cells from induced sputum or bronchioalveolar lavage (BAL) samples yields much less cells (< 2000) (Table 3), but these methods are completely non-invasive, and can be performed even in neonates [71]. Interestingly, despite similar initial cell numbers, cell cultures from induced sputum have lower culture initiation rate, than cultures established from BAL (Table 3). Successful primary HAE cultures can also be established from tracheal aspirates from intubated infants [74]. Such samples contain extremely low initial number of cells (< 100 cells/aspirate), but due to the fact, they can be collected multiple times a day, the rate of culture initiation is relatively high (Table 3).

### Sample- and donor-related factors

During the culture initiation, mucus should be avoided, as it poses a source of contamination to the cultures and can lead to the failure of culture [32]. In the case of CF patients, specific cocktails of antibiotics (and sometimes also mucus-releasing factors) are employed at the beginning of the culture, to ensure proper removal of mucus and sterility of the culture from the start [78, 79]. Moreover, collected cells, no matter the number, should be in the best possible condition. A marker of a good sample condition is the presence of MCC cells, as they often first react to airway insults [80]. Studies have shown, that culture success rate in ALI setting is ~ two-fold lower for samples, which do not contain MCC cells in the initial biopsy (OR = 2.18, [1.50–3.16], p < 0.001) [80]. Outcome of the culture depends also on several donor-related factors, such as lifestyle and used medications. It has been observed that frequent inflammation and smoking reduce the proliferative potential of epithelial stem cells, and yield differentiated epithelium with the smaller number of club cell progenitors expressing SCGB1A1 [9]. The use of inhaled corticosteroids or long acting β-agonist should be avoided, to increase the culture success [80] (OR =  ~ 0.62–0.64; *p* = 0.01 or p = 0.02, respectively). Also some donor’s disorders (e.g. dry nose) can cause considerable difficulties in collecting sufficient number of cells for starting an effective culture (Bukowy-Bieryllo, unpublished observation). Furthermore, care should also be taken, when collecting the primary HAE cells, as local anesthetics and some inhaled drugs (e.g. lidocaine or hypertonic saline) can significantly reduce the cilia motility (lidocaine being least toxic) [81–83], and should thus be avoided.

### Tissue source

Another factor, which needs to be considered when planning experiments using primary airway cell culture, is the airway region, from which the tissue originates. Traditionally, the majority of studies use distal airway cells (bronchial or tracheobronchial epithelial cells) to initiate the culture, due to their established culture methods and availability in patients with respiratory disorders (regular bronchoscopies). However, cultures of cells from proximal airway (nasal epithelium, HNE) are gaining popularity, as an alternative, much less-invasive tissue source. Recent studies have shown, that the choice of one of these tissues can have profound effects not only on the culture yield, but also on the results of the experiments performed on these cultures.

First of all, the replicative lifespan of PSE cells isolated from the proximal and distal airway might not be equal. It has been observed, that CRC HAE bronchial cells co-cultured with feeder cells could divide for at least 15 passages [50, 69]. When HNEs were grown in the same CRC regimen, they showed a drop in cell growth already at P2 [27]. It has been speculated that the reduced BCs number, capable of proliferating, present in the sample, was responsible for this difference [27].

The difference between proximal and airway cells is also visible on the gene expression level. Despite an overlap in the gene expression profile between native HTEC and HBEC primary cells, the HAE from different airway regions have distinct molecular signatures [84]. Different gene expression profiles were also observed between HNE and human tracheobronchial cells [58].

There are contradicting reports regarding the differences in cellular physiology of the PSE cells from proximal and distal airway. Some studies have observed reduced IL-6 secretion and increased inflammatory response after cigarette smoke stimulation in HNEs, compared to HBEs[8]. Other studies have not observed such differences [85].

In any case, these observations mean that, when planning an experiment using primary airway epithelial cells, care should be taken when choosing the tissue source and the culturing platform. Moreover, to obtain reliable results, a careful validation of the tissue composition and physiology of the differentiated tissue is necessary.

## Conclusions

The recent development of CRC HAE culturing method, which increased the proliferative efficiency of the primary HAE epithelial cultures, has allowed to obtain high numbers of HAE cells, which can be genetically modified, cryopreserved and can even be used for tissue engineering. However, despite the progress made in increasing the HAE cell proliferative lifespan and the ability to differentiate, the CRC HAE model is still not perfect. Further improvements should be made to increase its robustness and replicability. This includes a thorough comparison of the existing CRC culturing types (CRC HAE mono- and co-culture regimens), in order to assess their similarity to the native HAE epithelium in terms of physiology, which will determine their suitability for particular types of experiments. Improved knowledge on the factors influencing the differentiation and functioning of HAE, including the cross-talk between ECM and different tissue types, such as fibroblasts, will contribute to that progress. Identification of the factors influencing donor-to-donor culture variability will help to predict the culture success, leading to a further increase in CRC culture replicability.

Of course, CRC cultures can in some aspects be replaced by other existing HAE culturing methods, such as 3D organoid cultures or iPSC-derived HAE cells. In all cases, the limitations of these different HAE culture types should always be carefully considered, before choosing the most appropriate HAE cellular model for particular study in airway research.

Considering the existing drawbacks of the iPSCs and organoid culture (reduced preservation of epigenetic marks in iPSCs culture or the technical difficulties related to organoid size control), there is a large chance that primary CRC HAE cultures, including mono- and co-cultured HAE cells, will still stay around for some time in respiratory research.

## Data Availability

Not applicable.

## Abbreviations

3T3: Albino Swiss mouse embryo fibroblasts feeder cell line
AECBM: Airway epithelial cell basal medium
ALI: Air-liquid interface
BAL: Bronchioalveolar lavage
BC: Basal cell
BEGM: Bronchial epithelial growth medium
BMPi: Inhibitor of the bone morphogenetic protein pathway
CF: Cystic fibrosis
CFTR: Cystic fibrosis transmembrane conductance regulator
COPD: Chronic obstructive pulmonary disease
CRC: Conditionally reprogrammed cell
CRISPR/Cas9: Clustered regularly interspaced short palindromic repeats-cas9 system
DLL-1: Delta-like protein 1
DMH-1: Dorsomorphin homolog 1, inhibitor of activin receptor-like kinase 2
ECM: Extracellular matrix
GTPase: Guanosine triphosphate (GTP) hydrolase
HAE: Human airway epithelial
HBE: Human bronchial epithelium
HNE: Human nasal epithelium
HTE: Human tracheal epithelium
hTERT: Telomerase reverse transcriptase, catalytic subunit of telomerase
iPSCs: Induced pluripotent stem cells
KSFM: Keratinocyte serum-free medium
LHC-9: Lechner's serum-free bronchial epithelial growth medium
MCC: Multi-ciliated columnar cell
MMP: Metaloproteinase
mTOR: Mammalian target of rapamycin kinase of the phosphatidylinositol 3-kinase-related family
NEB: Neuroendocrine body
Notch ICD: Notch intracellular domain
PAK1: P21 associated kinase
PCD: Primary ciliary dyskinesia
PSE: Pseudostratified respiratory epithelium
ROCK: Rho-associated coiled-coil–containing protein kinase
ROCKi: Inhibitor of the Rho-associated coiled-coil–containing protein kinases
SAGM: Small airway growth medium
SMAD: Human homologs of the Caenorhabditis elegans SMA ("small" worm phenotype) and Drosophila MAD ("Mothers Against Decapentaplegic") family of genes
TEER: Transepithelial resistance
